# A Convolutional Neural Network for Lentigo Diagnosis

**DOI:** 10.1007/978-3-030-51517-1_8

**Published:** 2020-05-31

**Authors:** Sana Zorgui, Siwar Chaabene, Bassem Bouaziz, Hadj Batatia, Lotfi Chaari

**Affiliations:** 8 Digital Research Centre of Sfax, Sfax, Tunisia; 9 Institut Mines-Télécom, CNRS, Paris, France; 10 Université de Sherbrooke, Sherbrooke, QC Canada; 11 Digital Research Centre of Sfax, Sfax, Tunisia; 12 University of Sfax, Sfax, Tunisia; 13 MIRACL and CRNS, University of Sfax, Sfax, Tunisia; 14 University of Toulouse, IRIT - INP-ENSEEIHT, Toulouse, France

**Keywords:** Reflectance Confocal Microscopy, Lentigo, CNN classification, InceptionV3

## Abstract

Using Reflectance Confocal Microscopy (RCM) for lentigo diagnosis is today considered essential. Indeed, RCM allows fast data acquisition with a high spatial resolution of the skin. In this paper, we use a deep convolutional neural network (CNN) to perform RCM image classification in order to detect lentigo. The proposed method relies on an InceptionV3 architecture combined with data augmentation and transfer learning. The method is validated on RCM data and shows very efficient detection performance with more than 98% of accuracy.

## Introduction

Reflectance Confocal Microscopy (RCM) [1] is a modality increasingly used in medical imaging like MRI (Magnetic Resonance Imaging) [2–4] or X-ray imaging [5]. In vivo RCM technique is easy to use during the patient examination and acquires high resolution skin images in a short time. This modality can be used to help dermatologists diagnose different skin diseases. However, it takes a long time for dermatologists to make full use of the possibilities of this technique for diagnostic purposes. Our work aims to develop a new tool to automate certain diagnostic steps required using deep learning [6]. On the other side, the lentigos are age spots that mainly appear on the hand or on the areas most frequently exposed to the sunlight. On the surface, they appear as a darker spot. Inside the skin layers, it is mainly at the level of the dermis-epidermis junction that the differences can be visible [7]. Therefore, the distinction of lentigos can be made using the RCM images. Several deep learning architectures, especially convolutional neural network (CNN) [5, 8] show great potential in medical imaging classification. In this paper, we propose a new 3D RCM image (2D + depth) classification method for lentigo detection. The method is based on a CNN on InceptionV3 architecture [9].

Until now, little works have been proposed for lentigo/healthy classification of RCM images. In [10], the authors perform a two-dimensional wavelet decomposition. Then a generalized Gaussian distribution was applied to the wavelet coefficients in order to perform a quantitative analysis assisted by a support vector machine (SVM) to classify RCM images obtaining an accuracy of 84.4%. Another approach in [11] explores a new unsupervised Bayesian algorithm for the joint reconstruction and classification of RCM images. The resulting algorithm for healthy and lentigo classification reached an accuracy percentage of 97%. Beside, the paper [12] automatically diagnosed lentigo by using three separate feature extraction methods like Wavelets, Haralick and CNN by Transfer Learning. The healthy/lentigo classification results reached an accuracy of 76%.

The present paper is organized as follows. Section 2 presents the problem formulation of lentigo diagnosis. Section 3 detailed the proposed lentigo detection method. Section 4 presents the experiment validation of our method. Finally, conclusion and some perspectives are drawn in Sect. 5.

## Related Work

### Lentigo Detection

**Fig. 1.**
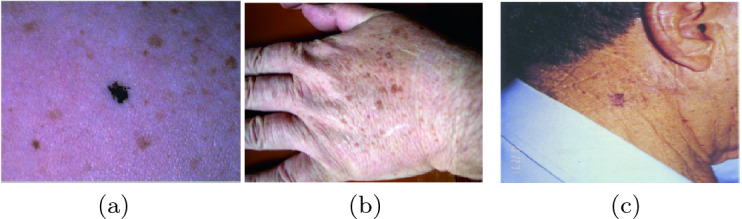
Lentigo simplex (a), Solar lentigo (b) and Lentigo maligna (c).

Lentigo is a lesion that occurs in the dermal epidermal junction between the dermis and the epidermis involving a high concentration of melanocytes in the dermal papillae walls. Most forms of lentigo are benign [13] like lentigo simplex as Fig. 1(a) and solar lentigo as Fig. 1(b). They are usually removed for cosmetic purposes. However, certain types such as lentigo maligna [14] as Fig. 1(c) may be harmful and must be removed.

Usually, lentigo is diagnosed using dermatoscopy [15]. Sadly, non-pigmented melanocytes with this modality can go completely unnoticed leading to complications in identifying the lesion contours with precision. Hispathology [16] is also used to confirm the diagnosis, but it can be inconvenient due to the fact that it is an in vitro technique involving performing a biospy from the pigmented areas. For these reasons, the RCM modality emerged to solve the problems encountered before. Therefore, this modality allows the expert to carry out a real-time 3D data acquisition and to facilitate the full observation of the biological structures in deformation over time. Due to all of these reasons, our approach is based on images acquired thanks to this modality. In [10, 11], the authors propose two RCM lentigo detection methods based on the statistical and Bayesian models [17] respectively. The methods have proved complicated and hard to implement. They require manual procedures like feature selection and data preparation. To this regard, we propose here a method for RCM image classification using a CNN architecture. Indeed, CNNs have proven their capacity to efficiently solve several complex problems in medical imaging.

### Convolutional Neural Networks

**Fig. 2.**
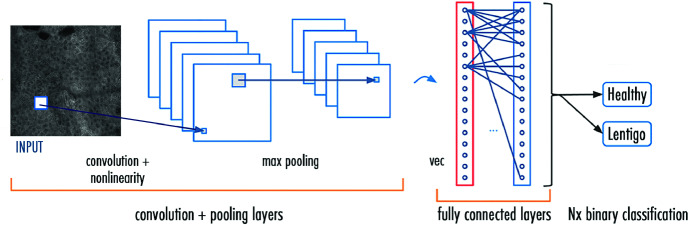
The CNN architecture model.

The CNN [8] is a deep learning architecture that is primarily used for image classification and object detection. Figure 2 displays a general CNN architecture, where one can easily identify the following layers:

- The convolutional layers: a key component of a CNN architecture, used for automatic feature extraction.
- The rectified linear units (ReLU): used after each convolutional layer. Each layer combines nonlinear layers and rectification layers to add nonlinearity to the system.
- The pooling layers: used for feature selection by maximum or/and average pooling.
- The fully connected layers: also known as dense layers receiving the flattened (1D) feature map. Usually, the final fully connected layer has the same number of output nodes as the class numbers.
- The Softmax function: calculates the probabilities of each target class over all possible classes. This function helps determine the target class for the given inputs.

## Proposed Method for Lentigo Detection

**Fig. 3.**
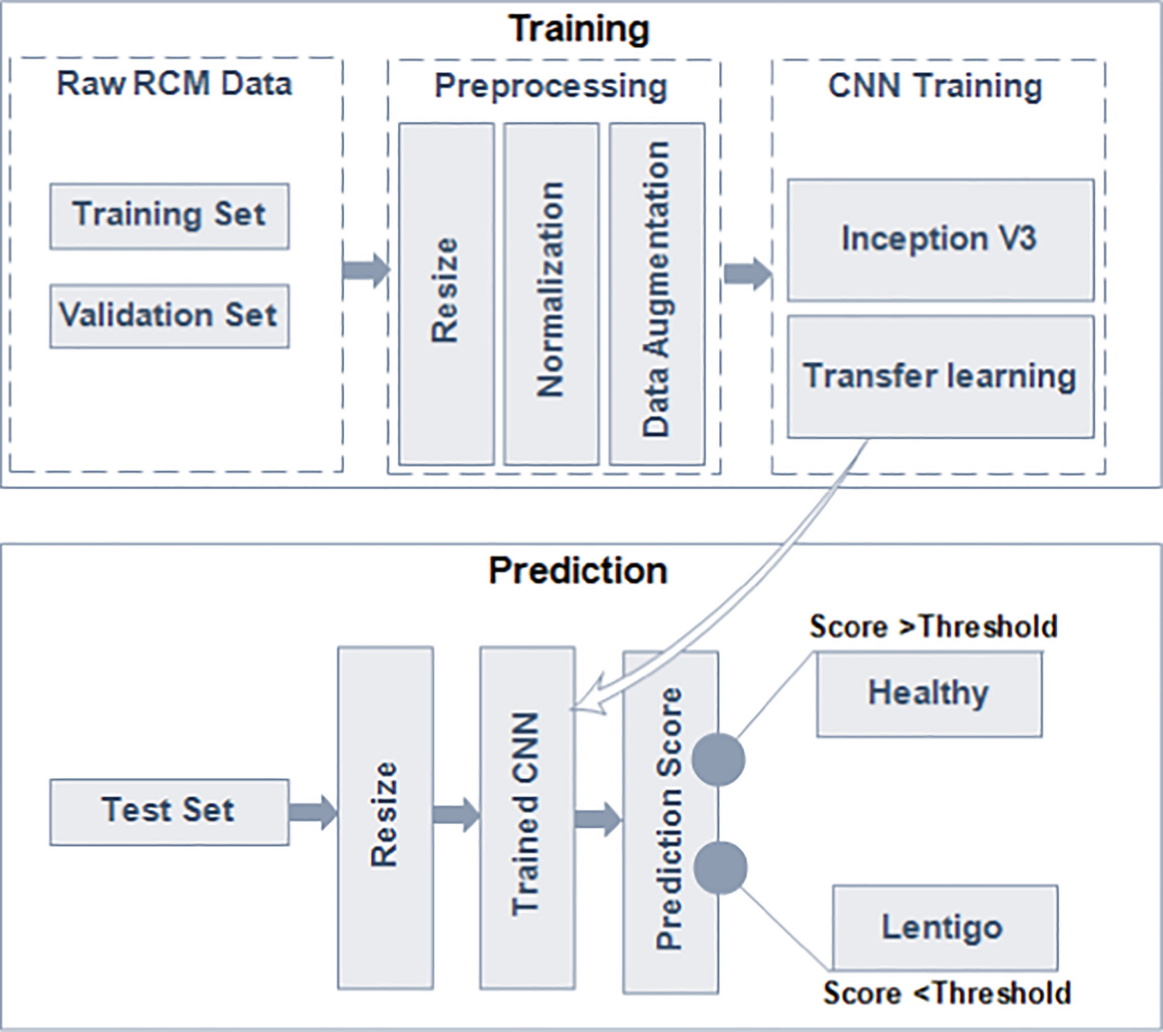
Pipeline of the proposed method.

The proposed method consists of classifying RCM images into healthy/lentigo classes using an InceptionV3 architecture. Our lentigo detection method combines the InceptionV3 model with other known deep learning techniques like transfer learning [18] and data augmentation [19]. Figure 3 presents the different steps used in the proposed lentigo detection method. The following subsections give detailed descriptions of each step.

### Data Preparation

The input RCM images for the training procedure combines two sets such as a 73% training set and a 14% validation set. The remaining 13% is dedicated to the prediction phase. In order to avoid overfitting, a validation set is added to our training phase because the non linear InceptionV3 model will possibly achieve 100% training accuracy and overfit.

### Data Preprocessing

In the first step of the preprocessing procedure, the RCM images of the training set are resized to fit in the InceptionV3 network. A normalization step is added to help the CNN better process the input images, in order that all feature values have the same range and the system needs only one global learning rate multiplier. Afterwards, the data augmentation step is proposed to improve our classification results. This step prevents accuracy decay and overfitting. In [20] the authors demonstrate the importance of data augmentation as a regulazier in the CNN classification model.

### InceptionV3 Model

**Fig. 4.**
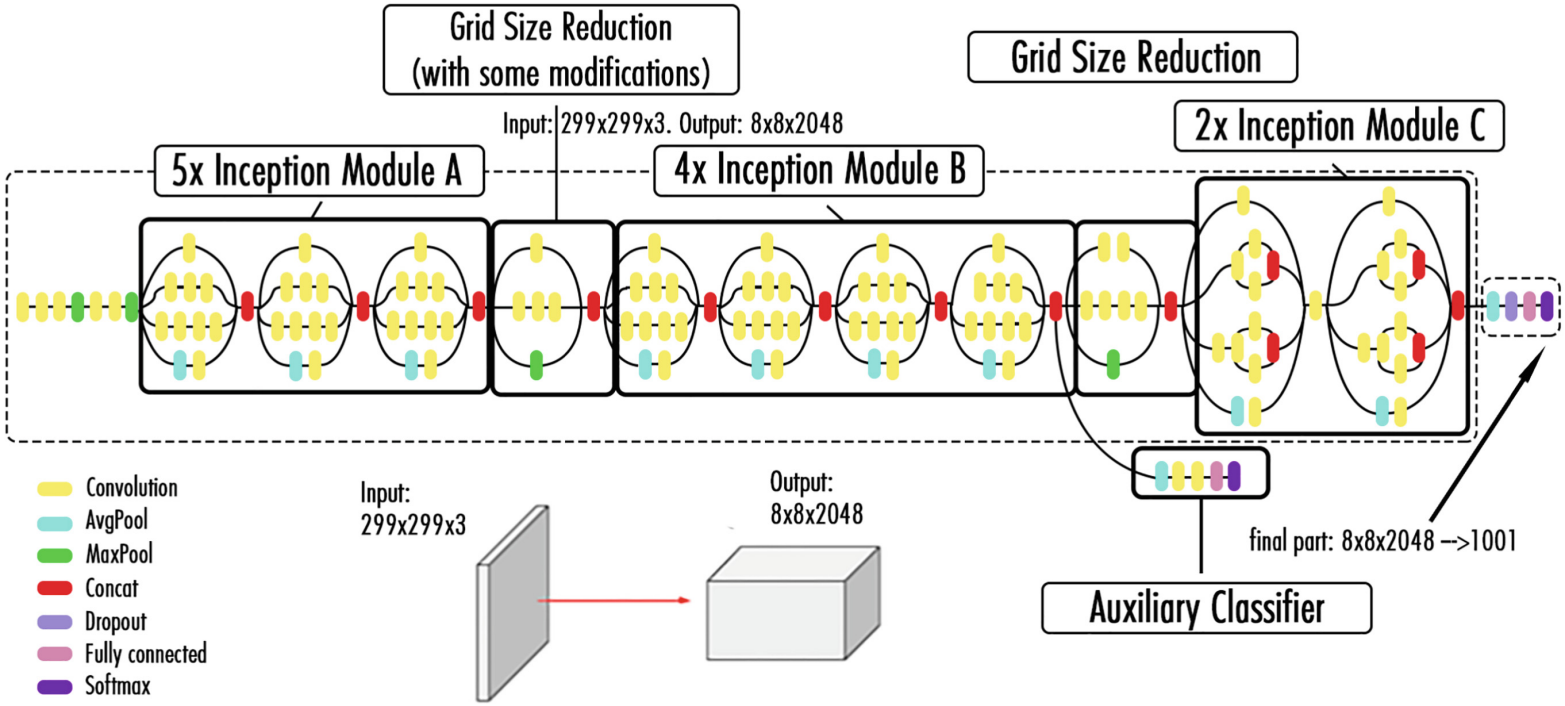
Architecture of the InceptionV3 model.

The InceptionV3 model is a complex heavily engineered network that considered a major breakthrough in CNN’s [9]. Before the current model, many common CNN’s claimed that stacking layers after layers is the only way to increase accuracy. However, this network suggested some solutions to improve accuracy and speed without piling many layers. As shown in Fig. 4, the InceptionV3 model consists of a combination of three main modules.

The first one (Module A) uses two smaller convolution layers ($3 \times 3$) to decrease the computational cost by reducing the number of parameters to improve performance. Module B divides each convolution layer of $n \times n$ size to two layers of $1 \times n$ and $n \times 1$ dimensions to have a less complex network. Finally, Module C reduces the representational bottleneck by expanding the filters in order to evade information loss. More upgrades are also proposed by the InceptionV3 network other than the smart factorization methods such as:

- RMSProp optimizer allows a faster convergence of the model thus allowing a higher learning rate.
- BatchNorm reduces the covariance shift and allows each network layer to learn a little independently of the others.
- Label Smoothing is a regularizing component applied to the loss formula to prevent overfitting.

The InceptionV3 network is 42 layers deep. Therefore, the computational cost is just around 2.5 higher than GoogLeNet’s [21]. In addition, the inception modules are a novel and popular concept due to their smaller convolutions, which explains the reduction in the number of parameters. The InceptionV3 model gathers more information without impacting the computational speed thanks to its depth and the various kernel sizes used in the convolution operations.

### Transfer Learning

As shown in Fig. 3, transfer learning [22] is proposed in order to ensure better performance of the model. The model needs lots of labeled images to be capable of solving complex problems. This has proved to be challenging especially when the available dataset is small. Transfer learning is a deep learning method, in which a model developed for a task is reused for a second task. This technique uses pre-trained models as a starting point for other medical imaging tasks given the vast computational and time resources required to develop CNN models on these problems.

### Prediction Model

In the prediction phase, the RCM images test set are resized and provided to the trained CNN. Our system calculates a prediction score for each test image after resizing it and compares it with the threshold T equal to 0.5. The threshold value is chosen that way due to the fact that we are performing a binary classification. The classification condition is as follows: if the predicted score (PS) value of the image test is lower than T then this RCM image is classified as lentigo and conversely.

## Experimental Validation

This section evaluates the validation of the proposed lentigo detection method on real RCM data. In our work, the dataset is provided from Lab. Pierre Fabre. In this experiment, the data include 428 RCM images which high spatial resolutions and annotation on each image into two healthy and lentigo classes. The images were acquired with a Vivascope 1500 apparatus. Each RCM image shows a field of view of $500 \times 500$ $\upmu$m with $1000 \times 1000$ pixels. A selection of 45 women aged 60 years were recruited. All participants have offered their informed consent to the RCM skin test. We split these data into three main sets:

- A 314 images training set divided into two classes of 160 healthy images and 154 lentigo images.
- A validation set of 60 images, divided equally between two classes of lentigo and healthy. The validation set has been added to evaluate our training procedure. The main objective is to prevent over-fitting.
- A 54 RCM images testing set divided equally for healthy and lentigo classes.

Our classification method based on the InceptionV3 network is build using the Keras library. The InceptionV3 model is configured to accept the greyscale RCM images. As initialization, all RCM images were resized into new dimensions of $299 \times 299$ pixels and rescaled to help CNN processing. The parameter values of data augmentation step are presented in Table 1. The shear, zoom and translation ranges vary from 0 to 1. We choose the value of 0.2 for each to enrich the dataset without altering the image main features and confusing the system. The rotation range varies to 0$^\circ$ from 180$^\circ$ and a small rotation angle was proposed for the same reasons.

**Table 1.** Data augmentation parameters.

Parameter	value
Shear	0.2
Zoom	0.2
Rotation degree	20$^\circ$
Horizontal translation	0.2
Vertical translation	0.2

**Fig. 5.**
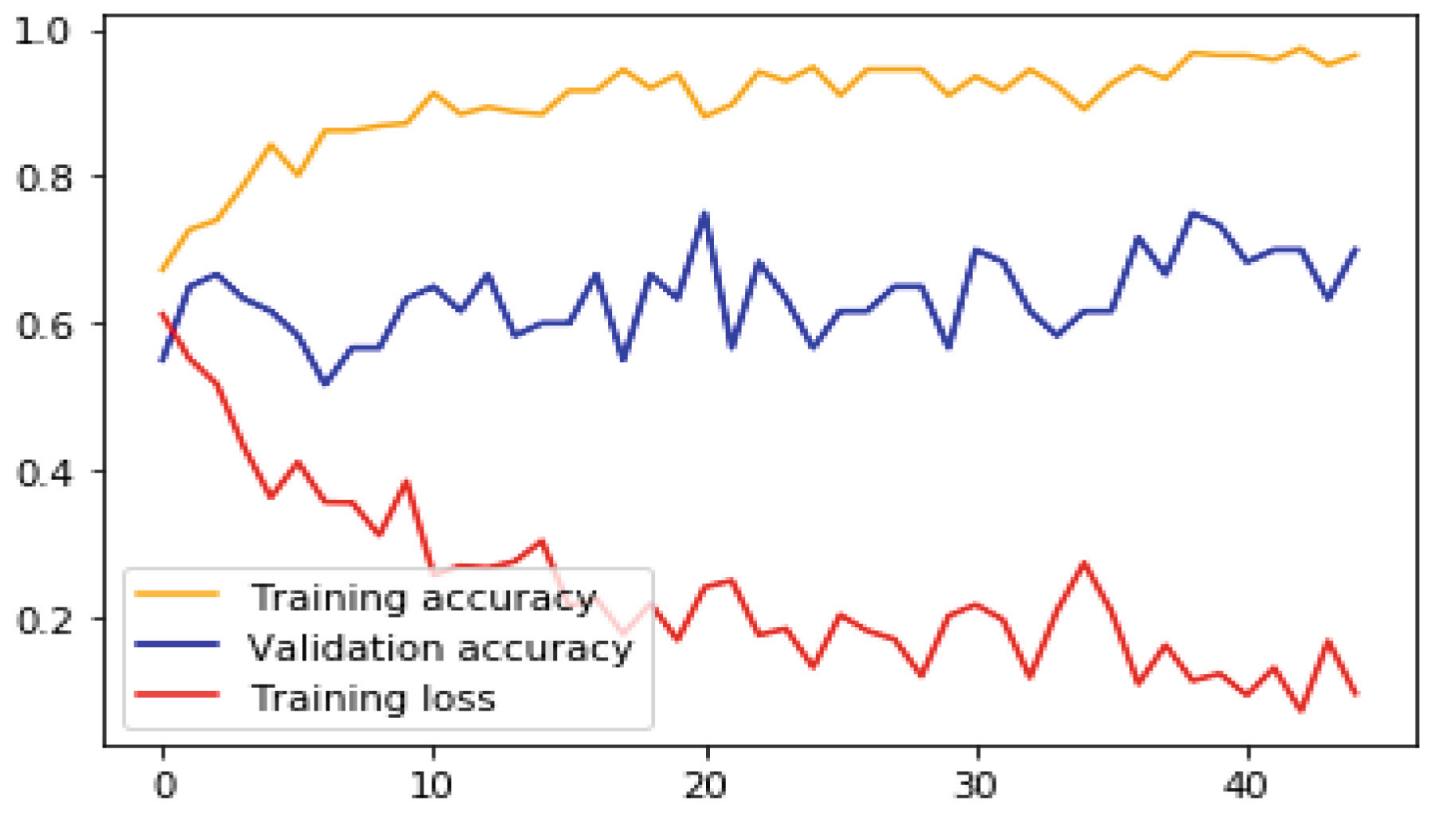
Proposed method accuracy graph and loss graph for training and validation sets after each epoch.

Figure 5 displays the accuracy curves of the training and validation sets, as well as the training loss. The accuracy curves suggest that our system converged after 40 epochs. The system reached an accuracy value of 94% for training and 69% for validation. Hence, the reported values indicate that our system learns well without over- or under-fitting.

The performance of the proposed method is indicated by the test set according to the ability to correctly diagnose the provided skin tissues. The reported values in Table 2 indicate the performance of our classification method. Therefore, 53 out of 54 images test set were correctly classified with an accuracy of **98,14**%.

**Table 2.** Confusion matrix.

	Lentigo	Sane
Lentigo	27/27 = 100% (TP)	1/27 = 3,7% (FN)
Sane	0/27 = 0% (FP)	26/27 = 96,3% (TN)

In Table 2, TP, TN, FP and FN represent respectively true positives, true negatives, false positives and false negatives. Based on the confusion matrix, Accuracy, Precision, Specificity, Recall and F-score values are reported in Table 3. All the mentioned measures indicate a good performance of the proposed method with values equal or very close to one.

**Table 3.** Quantitative evaluation of the proposed method performance.

**Accuracy**	(TP + TN)/(TP + TN + FP + FN)	0.98
**Precision** (P)	TP/(TP + FP)	1
**Specificity**	TN/(FP + TN)	1
**Recall** (R)	TP/(TP + FN)	0.96
**F-score**	(2 $\times$ P $\times$ R)/(P + R)	0.97

**Fig. 6.**
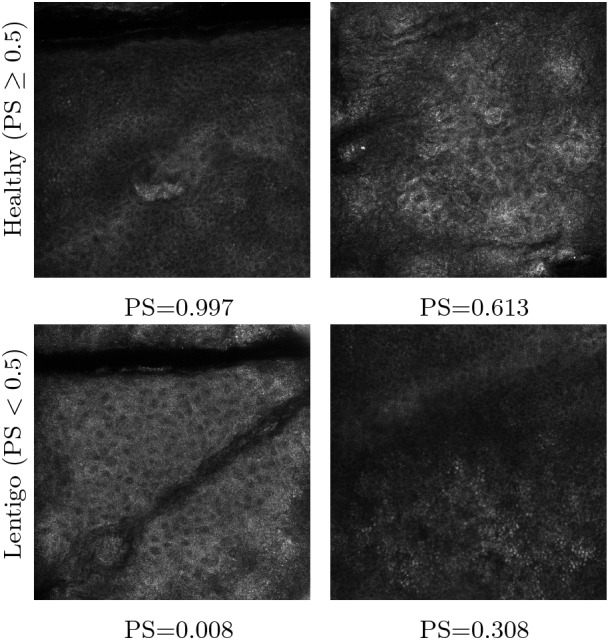
Correct classification examples of RCM images for Healthy and Lentigo patients classified by the proposed method.

Figure 6 presents four correct classification examples of RCM images from the test set. The reported values shown with each test image indicate the prediction score (PS). The displayed images correspond to different PS ranges. We can notice that the model performs well both for images with PS close to 0 or 1, but also for images with PS close to 0.5 (images (b) and (d)).

**Fig. 7.**
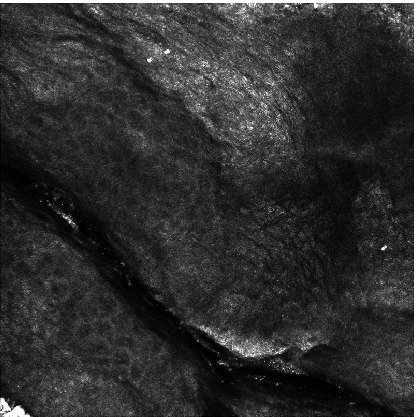
The only false classification (PS = 0.0005).

Figure 7 shows the only image wrongly classified using our proposed method. This image shows some type of skin deformation similar to the changes the skin undergoes due to lentigo. Hence, the network interpreted it as a lentigo lesion.

For the sake of further evaluation, we compare the accuracy of the test with related works that used the same dataset. The reported values in Table 4 show that our model outperforms in comparison with the other methods. Specifically, we compare our results with those reported in [10] where the authors used a Statistical model combined with an SVM classifier and [11] where the authors use an unsupervised Bayesian approach.

**Table 4.** Comparison performance with state of the art methods.

Lentigo detection method	Accuracy
Halimi et al. 2017 [10]	84.4%
Halimi et al. 2017 [11]	97.7%
Proposed method	**98,14**%

## Conclusion

In this paper, we proposed a new method to classify RCM images into healthy and lentigo skins. This method is based on the InceptionV3 CNN architecture. The network was trained with a dataset of 374 images and tested on 54 images of different stacks and depths. The suggested CNN method shows huge potential and very promising results. In future work, we will focus on applying the proposed approach to larger datasets and comparisons to other deep architectures.
